# Multimodal single‐cell analysis provides novel insights on ankylosing spondylitis in females

**DOI:** 10.1002/ctm2.1066

**Published:** 2022-10-07

**Authors:** Hsin‐Hua Chen, Jing‐Rong Wang, Hsiao‐Ni Sung, Wen‐Cheng Chao, Kuan‐Ting Liu, Fang‐Ping Lin, Tai‐Ming Ko

**Affiliations:** ^1^ Division of General Medicine Division of Allergy, Immunology and Rheumatology, Department of Internal Medicine Taichung Veterans General Hospital Taichung Taiwan; ^2^ School of Medicine National Yang Ming Chiao Tung University Taipei Taiwan; ^3^ Department of Biological Science and Technology National Yang Ming Chiao Tung University Hsinchu Taiwan; ^4^ Department of Critical Care Medicine Taichung Veterans General Hospital Taichung Taiwan; ^5^ Institute of Bioinformatics and Systems Biology National Chiao Tung University Hsinchu Taiwan; ^6^ Institute of Biomedical Sciences Academia Sinica Taipei Taiwan; ^7^ Center for Intelligent Drug Systems and Smart Bio‐devices (IDS^2^B) National Yang Ming Chiao Tung University Hsinchu Taiwan; ^8^ School of Pharmacy, College of Pharmacy Drug Development and Value Creation Research Center Kaohsiung Medical University Kaohsiung Taiwan

1

Dear Editor,

Ankylosing spondylitis (AS) is a chronic rheumatic disease that causes disability and severe impairment in quality of life, especially in females.^1^, ^2^ Based on clinical observations, diagnosing female patients with AS is challenging because of the minor radiation damage. However, little is known about how large heterogeneous circulating immune cells are involved in AS development in females.^3^ To overcome these limitations, single‐cell‐resolved gene expression profiling was used to characterize the immune cell status profile in the blood of female AS patients.

To identify targets specific to female AS in heterogeneous cell populations, single‐cell RNA sequencing (scRNA‐seq) was performed on peripheral blood mononuclear cells (PBMCs) of three female patients with AS and five sex‐matched (female) healthy control individuals (Figure 1A and Table S1). For visualization, scRNA‐seq data of PBMCs were integrated for unsupervised dimension‐reduction clustering using uniform manifold approximation and projection (Figure 1B). Immune cell features were identified (Figure 1B,C), and the expression profile of the immune marker genes in each cluster was confirmed (Figure 1D). Furthermore, we observed a dynamic change in the composition of PBMCs from female AS patients (Figure 2A and Table S2).

**FIGURE 1:**
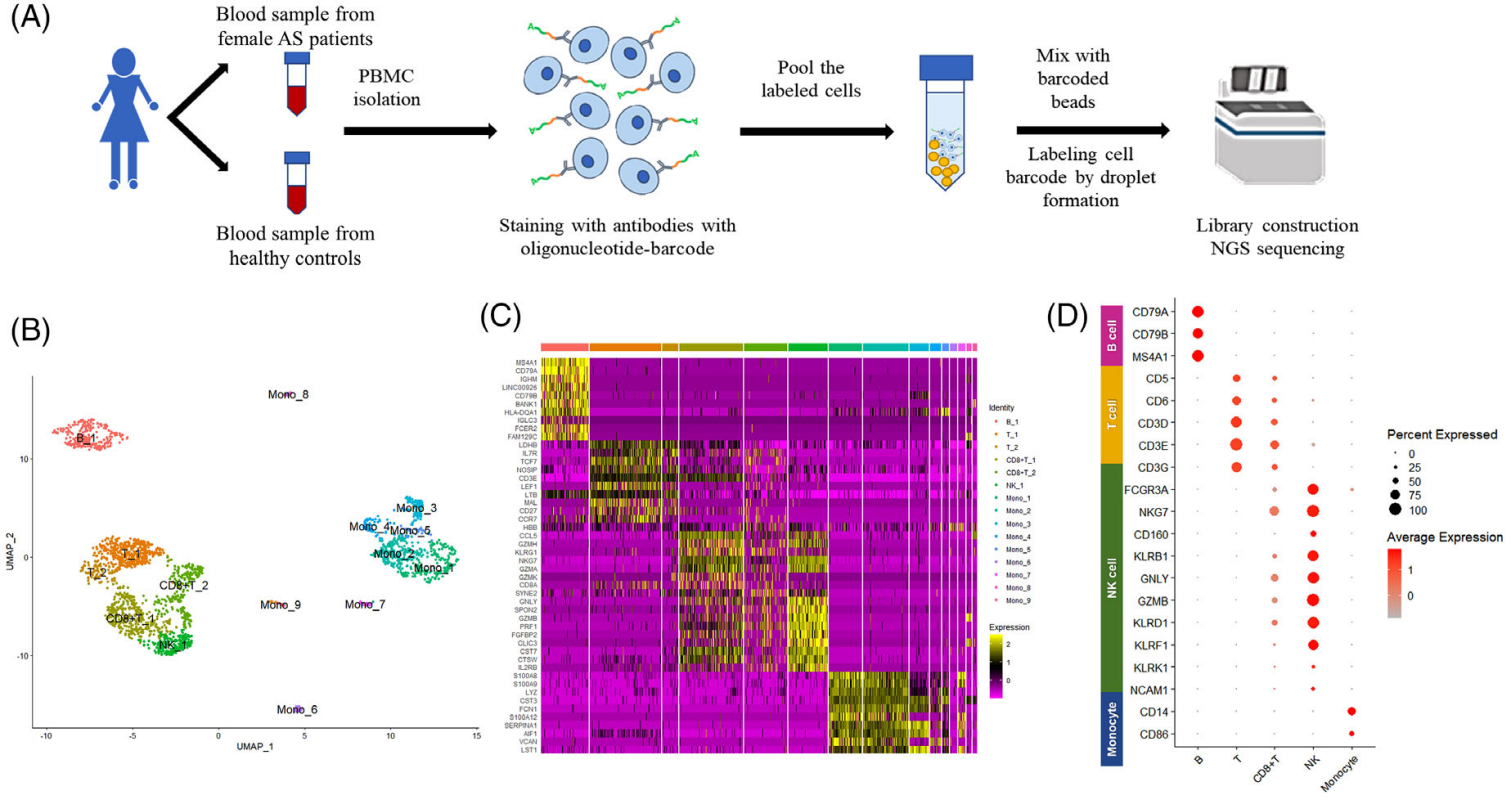
Study design, experimental workflow and immune composition of PBMCs. (A) Blood PBMCs were isolated from female AS patients and gender‐matched healthy controls. The  DNA‐barcoded antibodies were incubated with each sample. The labelled cells were loaded into the micro‐fluid system, and single droplets which contained single gel beads were constructed. After lysing the labelled cells, NGS libraries were constructed by cDNA synthesis. Single‐cell analysis was performed after sequencing by a high‐throughput platform – NovaSeq (Illumina) and preprocessing the raw data. (B) Composition of the PBMCs of AS patients (*n* = 3) and healthy controls (*n* = 5). Cell annotation was constructed using an auto‐classifier, singleR. (C) Heatmap of specific feature gene expression profile of each cell subpopulation. (D) Immune cell marker gene expression of each cell subset. The immune cell marker genes from feature genes of each cell subpopulation were selected using the CellMarker database. The result of cell annotation was verified using the auto‐classifier.

**FIGURE 2:**
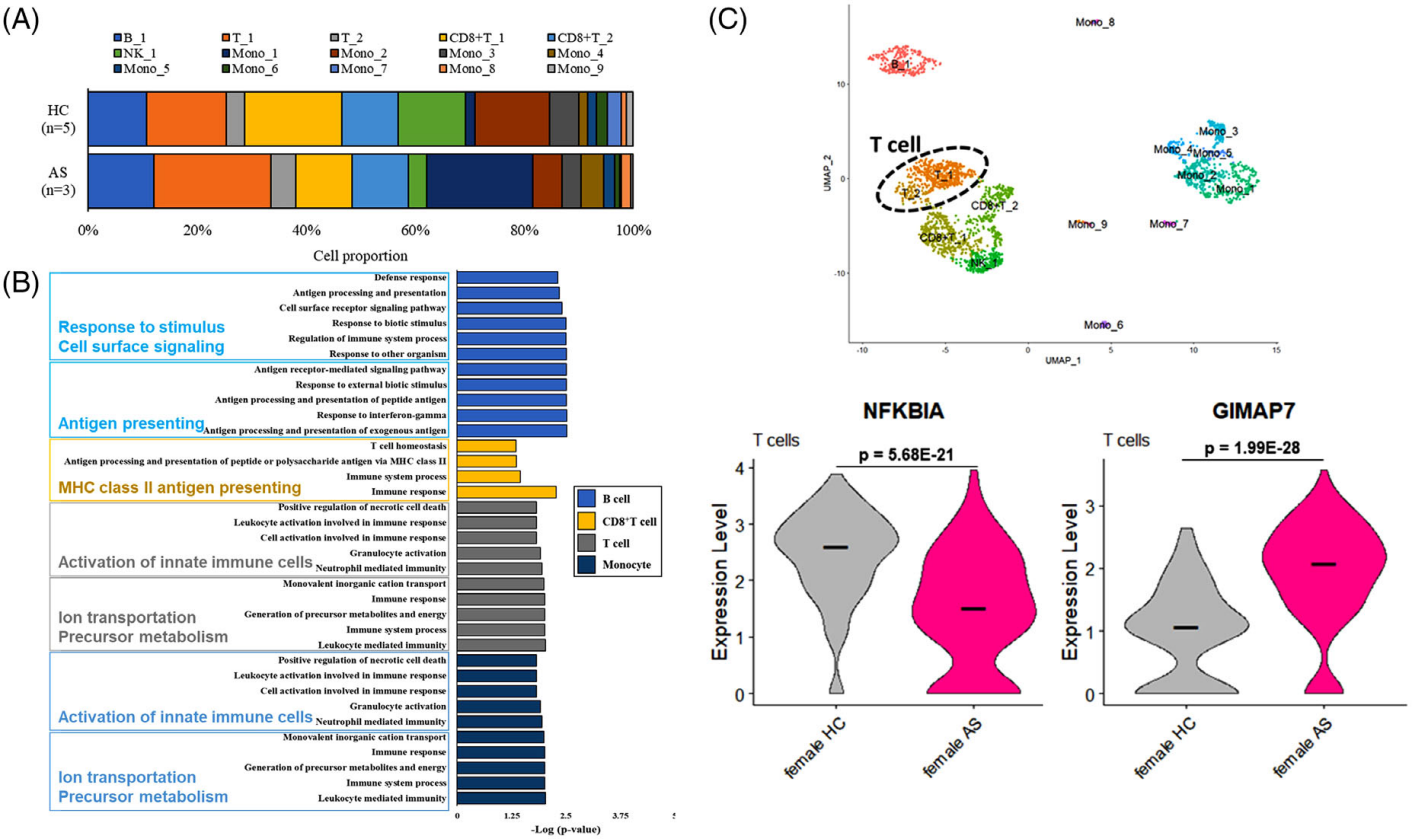
Single‐cell bio‐signatures in the T cells from female AS patients. (A) Composition of each cell cluster of the female AS patients (*n* = 3) and the gender‐matched healthy controls (*n* = 5). (B) Upregulated biological pathways of each cell group via gene ontology (GO) analysis and differential expressed genes of the female AS patients compared to the gender‐matched healthy controls. (C) The indicated gene expression (*NFKBIA*, *GIMAP7*) of the T cell population (including T_1 and T_2) between the female AS patients and the gender‐matched healthy controls.

To explore disease‐associated expression features, differential expression analysis was performed to determine the significance‐filtered differentially expressed genes (DEGs) in patients and healthy controls (Table S3). The gene ontology pathway analysis of each cell type was conducted based on the differential expression analysis between the patient group (*n* = 3) and the healthy control group (*n* = 5) (Figure 2B).

To further identify critical targets in female patients with AS, feature selection was performed using multiple filters that collected the common DEGs from the strict‐significance‐filtered (*p*< .001) DEGs of each cell type in the patient group (*n* = 3) compared to the healthy group (*n* = 5) (Figure S1 and Table S3). Among the 167 DEGs in female AS patients, we identified two common cell‐type‐specific DEGs, *NFKBIA* and *GIMAP7*, shared in all female AS patients using multiple filters. Compared with healthy controls, *NFKBIA* was significantly downregulated in the T cells of female AS patients (*p* = 5.68E‐21), while *GIMAP7* was significantly upregulated (*p* = 1.99E‐28) (Figure 2C).

The pseudo‐time trajectory of the T cells in the female patients showed a completely opposite differentiation direction to that obtained for the gender‐matched healthy controls. From pseudo‐time‐1 to pseudo‐time‐5, the T cells of state‐7 and state‐8 were only present in female AS patients. In addition, the cells of state‐7, state‐8 and T cells of female AS patients showed upregulation of *GIMAP7* and downregulation of *NFKBIA*. Furthermore, male AS patients showed no NFKBIA downregulation (Figure S2). These data confirmed the presence of female AS‐specific T cells and delineated their transcriptomic features (Figure 3A).

**FIGURE 3:**
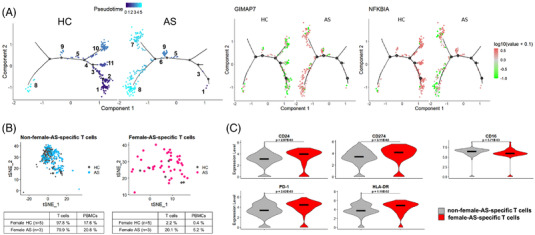
Uncovering unique features of T cells obtained from AS patients in females. (A) Trajectory analysis and differential gene expression of the T cells from the female AS patients and the gender‐matched healthy controls. Eleven trajectory states were found in the T cells. Different cell trajectory states were enriched in the two conditions. Cells of states 6–9 were enriched in the T cells of the AS patients, the remaining states were enriched in the healthy controls. The two differentially expressed genes, *NFKBIA* and *GIMAP7*, showed differential expressions in the two conditions. *GIMAP7* was highly expressed in the T cells of the patients, while *NFKBIA* was highly expressed in the healthy controls. (B) The proportion of non‐female‐AS‐specific and female‐AS‐specific T cells in the PBMCs of the female AS patients and the gender‐matched healthy controls. The proportion of non‐female‐AS‐specific cells showed fewer differences between AS patients and the healthy controls. The proportion of the female‐AS‐specific cells was at least 10 times higher in the T cells and PBMCs of the female AS patients than in the gender‐matched healthy controls. (C) Cell surface protein expression in female‐AS‐specific cells compared to that in the non‐female‐AS‐specific cells in the T cells. Differential analysis of the surface protein expression depicted the upregulation of CD24, CD274, HLA‐DR and PD‐1 and the downregulation of CD16 in the female‐AS‐specific cells.

Based on the differential analysis and pseudotemporal ordering, the *GIMAP7* ^+^ *NFKBIA^−^* T cells were selected and defined as female AS‐specific T cells. We found a higher proportion of female AS‐specific T cells in the patient group than in the healthy controls (Figure 3B). In the cellular indexing of transcriptomes and epitopes (CITE‐seq)^4^ experiments, differential analysis of surface protein expression between female AS‐specific T cells and non‐female AS‐specific T cells showed significant upregulation of CD24 (*p* = 2.97E‐03), CD274 (*p* = 3.11E‐02), HLA‐DR (*p* = 1.19E‐02) and PD‐1 (*p* = 2.02E‐03) in female AS‐specific T cells, and a significant downregulation of CD16 (*p* = 5.71E‐03) (Figure 3C). Moreover, female AS‐dominant paired T cell receptor clonotypes and their amino acid sequences were identified (Figure S3).

To identify the origin of the female AS‐specific T cells, four cell–cell interactions (CCIs) that were only present in the patients were selected using the CellChat algorism^5^ (Figure S4 and Table S4). Based on the selection criteria (Supplementary Methods), the secreted signalling communication of vascular endothelial growth inhibitor (VEGI) was filtered out and assumed to be a potential signalling pathway (ligand‐receptor contribution: TNFSF15–TNFRSF25) of female AS‐specific T cells. Among the sender cell groups, NK cells conveyed the maximum VEGI signalling to T cell subset‐2. This implied that female AS‐specific T cells were probably activated by NK cells via the VEGI signalling pathway in patients with AS (Figure 4A,B).

**FIGURE 4:**
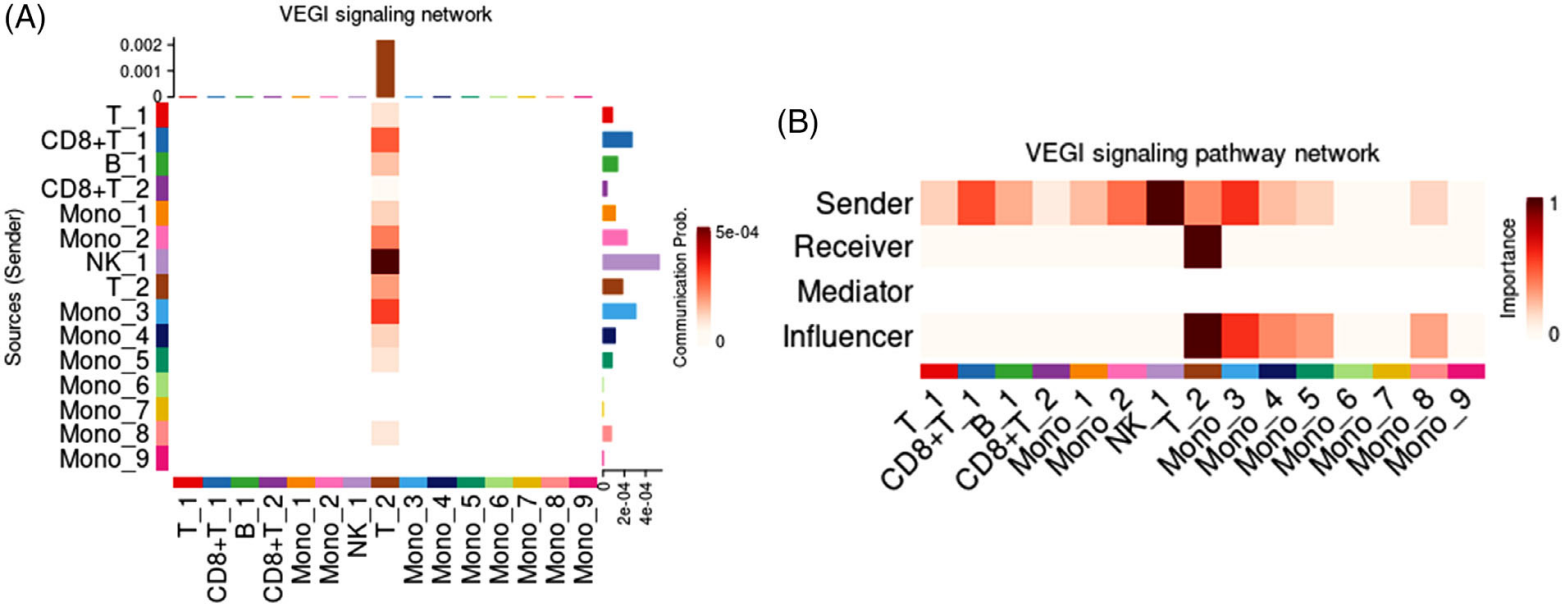
Cell–cell interactions in peripheral immune cells of AS female patients. The signalling of VEGI interaction between peripheral immune cell subpopulations was identified as a female‐AS‐specific cell communication by the CellChat algorithm (Figure S4). (A) Interaction heatmap summarizing net outgoing and incoming signals of VEGI interaction in respective cell types. NK cells, partial monocyte subsets (Mono_2 and Mono_3) and CD8^+^ T cells (CD8^+^T_1) convey the signalling to T cell subset‐2 (T_2) that gets converted into potential VEGI signalling sender (influencer). The VEGI signalling interaction between NK subset‐1 (NK_1) and T cell subset‐2 (T_2) shows the maximum communication probability (Prob_Com) than other cell populations (c = .053%, *p*‐value <.05 ). (B) Defining the roles of each peripheral immune cell subpopulation in the intercellular VEGI communication networks. The signalling of VEGI was dominantly released from the NK cells and transferred to the T cell subset‐2 (T_2).

AS displays clinical heterogeneity and complex blood transcription characteristics that support clinical heterogeneity.^6^ Sex differences in the neuroimmune interface functions may be responsible for the sex differences in the clinical manifestations, which can have important implications for AS. In the current study, multimodal single‐cell analysis was used to profile PBMCs from female patients with AS. These valuable datasets and results from the single‐cell comprehensive analysis, including cell composition, transcriptional changes, immune repertoire profiling, CCIs and pseudotemporal cell trajectory, provide new insights into the pathogenesis of AS. Here, we found that specific T cells (*GIMAP7^+^* and *NFKBIA^−^*) were polarized during AS in females (Figure S5). GTPases of the immune‐related protein family (*GIMAPs*) are mainly expressed in immune cells and are related to immune functions, such as peripheral lymphocyte apoptosis and T helper cell differentiation.^7^ The NF‐κB transcription factor family, including *NFKBIA*, regulates various aspects of T cell development, activation, differentiation and survival.^8^ Furthermore, a potential trigger of VEGI (TNFSF‐15) signalling, an endogenous negative regulator of angiogenesis,^9^ was identified in the development of pathogenic T cells by cell–cell communication analysis, which may also be correlated with the induction of proinflammatory cytokines in AS.^10^

As the number of patients was relatively small, future studies will benefit from increasing the sample size to better depict the differences between individuals and capture the full range of disease severity. The results of this study lay the foundation for these efforts.

In summary, our work has determined that specific T cell populations in the blood can be used as signatures for female patients with AS. Associated cell‐surface markers of these specific populations can potentially be applied to detect cell status, thereby providing the possibility of early monitoring in female individuals at risk of developing AS. Additionally, an upstream mechanism was discovered that regulates the function of T cells. These findings provide a new way to overcome the limitations of determining the potential immune response characteristics of patients with AS. They also offer new solutions for the development of more specialized treatments for AS in females.

## Supporting information

Supp information
