# Supplementary material for: Multimodal single‐cell analysis provides novel insights on ankylosing spondylitis in females
Source: Clin Transl Med. 2022 Oct 7;12(10):e1066. doi: 10.1002/ctm2.1066 (PMC9547115; doi:10.1002/ctm2.1066)
Supplement: Supplementary file 1 — Supp information [file CTM2-12-e1066-s001.docx]

**Table S1. Demographics and clinical characteristics.**

| **Patient** | **Sex** | **Symptom onset age** | **Patient global** | **Physician global** | **ESR mm/hr** | **CRP mg/dL** | **ASDAS_ESR** | **ASDAS_CRP** | **BASFI** | **ASAS_HI** |
| --- | --- | --- | --- | --- | --- | --- | --- | --- | --- | --- |
| AS-1 | F | 44 | 2 | 2 | 7 | 0.021 | 1.4 | 0.8 | 0.6 | 4 |
| AS-2 | F | 23 | 3 | 3 | 9 | 0.199 | 2.0 | 1.9 | 0.9 | 7 |
| AS-3 | F | 35 | 2 | 2 | 28 | 1.595 | 3.1 | 3.5 | 1.3 | 7 |
| average |  | 34 | 2 | 2 | 15 | 0.605 | 2.2 | 2.1 | 0.9 | 6 |
| AS-1 | M | 22 | 6 | 6 | 52 | 5.173 | 3.9 | 4.2 | 4.1 | 12 |
| AS-2 | M | 20 | 2 | 2 | 9 | 0.310 | 1.5 | 1.5 | 0.6 | 3 |
| AS-3 | M | 19 | 3 | 3 | 9 | 1.096 | 1.8 | 2.5 | 0.2 | 1 |
| AS-4 | M | 40 | 3 | 3 | 10 | 1.385 | 1.5 | 2.3 | 1.7 | 5 |
| average |  | 25 | 4 | 4 | 20 | 1.991 | 2.2 | 2.6 | 1.7 | 5 |

**Table S2. Cell population frequency in female AS patients and healthy controls**

| Frequency (% in PBMCs) | | |
| --- | --- | --- |
|  | Female | |
|  | AS (n=3) | HC (n=5) |
| B_1 | 12.1% | 10.8% |
| T_1 | 21.4% | 14.6% |
| T_2 | 4.6% | 3.4% |
| CD8^+^T_1 | 10.3% | 17.8% |
| CD8^+^T_2 | 10.3% | 10.3% |
| NK_1 | 3.4% | 12.4% |
| Mono_1 | 19.4% | 1.7% |
| Mono_2 | 5.5% | 13.7% |
| Mono_3 | 3.4% | 5.4% |
| Mono_4 | 4.2% | 1.7% |
| Mono_5 | 1.9% | 1.5% |
| Mono_6 | 1.0% | 2.1% |
| Mono_7 | 0.2% | 2.5% |
| Mono_8 | 1.7% | 0.9% |
| Mono_9 | 0.5% | 1.2% |

**Table S3. DEGs of each cell subpopulations in female AS patients.**

|  |  |  | Female AS patients (n=3) | HC  (n=5) |  |
| --- | --- | --- | --- | --- | --- |
| Cell type | Gene | avg_logFC | pct.1 | pct.2 | p_val_adj |
| B cell | *HLA-DRB5* | 1.13E+00 | 82% | 26% | 3.72E-16 |
| B cell | *CD180* | 7.71E-01 | 42% | 16% | 3.78E-03 |
| B cell | *LINC02397* | 6.69E-01 | 49% | 19% | 4.40E-03 |
| B cell | *HLA-DQA2* | 5.49E-01 | 31% | 8% | 2.61E-02 |
| B cell | *MT-CO1* | 5.34E-01 | 100% | 100% | 1.17E-03 |
| B cell | *CORO1A* | 5.01E-01 | 89% | 82% | 5.11E-03 |
| B cell | *ACTB* | 4.27E-01 | 97% | 98% | 2.67E-02 |
| B cell | *HLA-DQA1* | 3.45E-01 | 97% | 95% | 6.43E-03 |
| B cell | *RPS3A* | -2.94E-01 | 96% | 95% | 8.08E-07 |
| B cell | *RPL21* | -3.07E-01 | 86% | 95% | 3.34E-02 |
| B cell | *RPL41* | -3.08E-01 | 89% | 96% | 2.09E-03 |
| B cell | *EIF1* | -3.84E-01 | 97% | 98% | 2.59E-06 |
| B cell | *BTG2* | -5.12E-01 | 51% | 82% | 4.19E-03 |
| B cell | *PNRC1* | -5.17E-01 | 46% | 71% | 3.93E-02 |
| B cell | *HLA-B* | -5.27E-01 | 96% | 99% | 5.45E-12 |
| B cell | *CNOT1* | -5.37E-01 | 16% | 47% | 3.89E-02 |
| B cell | *LYN* | -5.37E-01 | 39% | 69% | 1.78E-02 |
| B cell | *SQSTM1* | -5.61E-01 | 37% | 69% | 9.89E-03 |
| B cell | *IER2* | -5.62E-01 | 78% | 94% | 6.66E-03 |
| B cell | *RBM3* | -5.88E-01 | 43% | 74% | 1.69E-03 |
| B cell | *TENT5C* | -6.13E-01 | 14% | 44% | 1.26E-02 |
| B cell | *YPEL5* | -6.22E-01 | 25% | 55% | 5.43E-03 |
| B cell | *IRF1* | -6.42E-01 | 30% | 62% | 1.74E-02 |
| B cell | *TAGAP* | -6.74E-01 | 53% | 76% | 1.12E-02 |
| B cell | *BTG1* | -6.78E-01 | 88% | 99% | 1.51E-07 |
| B cell | *NR4A1* | -6.84E-01 | 5% | 32% | 1.12E-02 |
| B cell | *DDIT3* | -6.85E-01 | 4% | 37% | 9.06E-05 |
| B cell | *PMAIP1* | -6.93E-01 | 17% | 51% | 2.77E-04 |
| B cell | *RBKS* | -7.44E-01 | 2% | 32% | 2.28E-04 |
| B cell | *FOS* | -7.44E-01 | 27% | 72% | 5.30E-07 |
| B cell | *KLF6* | -7.56E-01 | 37% | 81% | 3.66E-07 |
| B cell | *PCBP1* | -7.57E-01 | 41% | 79% | 1.65E-08 |
| B cell | *CSRNP1* | -7.65E-01 | 6% | 48% | 2.63E-07 |
| B cell | *PPP1R15A* | -8.04E-01 | 36% | 76% | 1.82E-07 |
| B cell | *PELI1* | -8.12E-01 | 13% | 49% | 1.62E-04 |
| B cell | *AC103591.3* | -8.62E-01 | 4% | 35% | 3.18E-04 |
| B cell | *ZFP36* | -8.83E-01 | 66% | 91% | 2.25E-09 |
| B cell | *H3F3B* | -9.24E-01 | 77% | 98% | 1.63E-13 |
| B cell | *NFKBIA* | -9.31E-01 | 69% | 93% | 2.50E-12 |
| B cell | *HERPUD1* | -9.47E-01 | 27% | 76% | 6.22E-10 |
| B cell | *DUSP1* | -1.02E+00 | 74% | 92% | 2.72E-11 |
| B cell | *JUNB* | -1.02E+00 | 74% | 95% | 1.75E-11 |
| B cell | *SLC2A3* | -1.11E+00 | 9% | 59% | 1.30E-10 |
| B cell | *DUSP2* | -1.12E+00 | 12% | 59% | 2.13E-08 |
| B cell | *TSC22D3* | -1.13E+00 | 52% | 89% | 2.20E-10 |
| B cell | *CXCR4* | -1.18E+00 | 30% | 81% | 2.84E-13 |
| B cell | *CD83* | -1.29E+00 | 28% | 82% | 1.29E-14 |
| B cell | *NR4A2* | -1.29E+00 | 3% | 53% | 5.19E-12 |
| B cell | *CD69* | -1.76E+00 | 27% | 91% | 7.01E-23 |
| T cell | *GIMAP7* | 9.43E-01 | 90% | 70% | 1.99E-28 |
| T cell | *LYZ* | 8.87E-01 | 33% | 7% | 2.59E-09 |
| T cell | *IL7R* | 7.09E-01 | 92% | 87% | 3.00E-10 |
| T cell | *GIMAP4* | 6.83E-01 | 67% | 40% | 1.51E-11 |
| T cell | *S100A9* | 5.75E-01 | 34% | 16% | 6.57E-03 |
| T cell | *CCR7* | 5.49E-01 | 74% | 63% | 1.12E-05 |
| T cell | *TUBA4A* | 5.25E-01 | 55% | 26% | 7.59E-09 |
| T cell | *DENND2D* | 4.96E-01 | 58% | 38% | 1.86E-05 |
| T cell | *GIMAP1* | 4.65E-01 | 63% | 48% | 6.42E-03 |
| T cell | *MYL12A* | 3.26E-01 | 94% | 95% | 5.96E-05 |
| T cell | *TBC1D10C* | 3.18E-01 | 72% | 60% | 2.48E-03 |
| T cell | *MT-CO1* | 3.14E-01 | 100% | 100% | 3.76E-03 |
| T cell | *GIMAP8* | 3.10E-01 | 19% | 5% | 1.23E-02 |
| T cell | *AL157402.2* | 3.09E-01 | 16% | 3% | 1.03E-02 |
| T cell | *EIF1* | -2.51E-01 | 99% | 100% | 9.56E-10 |
| T cell | *ATP5F1E* | -2.61E-01 | 88% | 98% | 6.65E-04 |
| T cell | *HLA-B* | -2.66E-01 | 99% | 100% | 2.16E-09 |
| T cell | *RPS29* | -2.70E-01 | 94% | 99% | 8.97E-26 |
| T cell | *CTLA4* | -2.88E-01 | 1% | 12% | 1.65E-02 |
| T cell | *C16orf87* | -2.97E-01 | 4% | 19% | 1.26E-02 |
| T cell | *CCDC59* | -3.03E-01 | 32% | 61% | 6.49E-04 |
| T cell | *BCL10* | -3.09E-01 | 8% | 26% | 1.69E-02 |
| T cell | *CNOT1* | -3.11E-01 | 13% | 37% | 2.42E-04 |
| T cell | *PELI1* | -3.12E-01 | 7% | 25% | 6.65E-03 |
| T cell | *CSRNP1* | -3.15E-01 | 12% | 38% | 6.82E-05 |
| T cell | *FOXP1* | -3.20E-01 | 60% | 80% | 2.39E-02 |
| T cell | *SAP18* | -3.23E-01 | 56% | 79% | 8.62E-04 |
| T cell | *MGAT4A* | -3.24E-01 | 41% | 65% | 4.78E-02 |
| T cell | *AC026979.2* | -3.27E-01 | 15% | 38% | 2.53E-03 |
| T cell | *RPL37A* | -3.29E-01 | 97% | 99% | 2.29E-14 |
| T cell | *HNRNPA0* | -3.32E-01 | 65% | 87% | 4.82E-04 |
| T cell | *TRA2B* | -3.36E-01 | 51% | 75% | 3.99E-03 |
| T cell | *SYF2* | -3.41E-01 | 34% | 59% | 1.83E-03 |
| T cell | *LEPROTL1* | -3.44E-01 | 56% | 79% | 4.29E-03 |
| T cell | *EIF4H* | -3.49E-01 | 32% | 58% | 1.41E-03 |
| T cell | *DUSP1* | -3.53E-01 | 73% | 87% | 4.11E-03 |
| T cell | *G3BP2* | -3.54E-01 | 37% | 59% | 3.21E-02 |
| T cell | *C6orf48* | -3.54E-01 | 22% | 54% | 6.86E-06 |
| T cell | *RPL37* | -3.55E-01 | 98% | 100% | 9.77E-25 |
| T cell | *GOLGA8B* | -3.67E-01 | 6% | 29% | 5.15E-06 |
| T cell | *PNRC1* | -3.73E-01 | 58% | 83% | 6.41E-05 |
| T cell | *RPS21* | -3.73E-01 | 99% | 100% | 2.78E-23 |
| T cell | *BTG2* | -3.74E-01 | 49% | 79% | 1.31E-06 |
| T cell | *HIST1H1E* | -3.74E-01 | 29% | 51% | 1.01E-02 |
| T cell | *RELB* | -3.81E-01 | 14% | 38% | 4.78E-04 |
| T cell | *SF1* | -3.82E-01 | 75% | 91% | 1.02E-06 |
| T cell | *RPL36A* | -3.85E-01 | 83% | 98% | 2.09E-12 |
| T cell | *SBDS* | -3.85E-01 | 44% | 72% | 9.62E-05 |
| T cell | *MT-ND3* | -3.90E-01 | 97% | 100% | 3.01E-14 |
| T cell | *RPL41* | -3.91E-01 | 98% | 100% | 9.40E-29 |
| T cell | *DDIT3* | -3.91E-01 | 6% | 28% | 2.65E-05 |
| T cell | *RPL38* | -4.11E-01 | 91% | 97% | 1.28E-14 |
| T cell | *YPEL5* | -4.12E-01 | 24% | 51% | 2.33E-04 |
| T cell | *RPS27* | -4.21E-01 | 98% | 99% | 1.50E-27 |
| T cell | *MT-ND6* | -4.50E-01 | 46% | 78% | 1.72E-07 |
| T cell | *TXNIP* | -4.70E-01 | 92% | 99% | 1.76E-10 |
| T cell | *RPL21* | -5.06E-01 | 95% | 99% | 6.25E-26 |
| T cell | *DNAJB1* | -5.74E-01 | 48% | 80% | 6.47E-11 |
| T cell | *H3F3B* | -5.97E-01 | 96% | 99% | 3.43E-26 |
| T cell | *TSC22D3* | -6.14E-01 | 54% | 84% | 1.38E-10 |
| T cell | *CD69* | -6.29E-01 | 55% | 84% | 7.54E-11 |
| T cell | *NFKBIA* | -7.18E-01 | 78% | 96% | 5.68E-21 |
| T cell | *CXCR4* | -7.27E-01 | 25% | 63% | 1.02E-12 |
| T cell | *PCBP1* | -8.58E-01 | 44% | 90% | 1.73E-28 |
| T cell | *TNFAIP3* | -9.09E-01 | 35% | 77% | 3.20E-19 |
| CD8^+^T cell | *LYZ* | 1.01E+00 | 37% | 8% | 1.63E-14 |
| CD8^+^T cell | *GIMAP7* | 9.21E-01 | 62% | 43% | 1.39E-08 |
| CD8^+^T cell | *HLA-DRB5* | 7.49E-01 | 28% | 10% | 9.44E-05 |
| CD8^+^T cell | *HLA-DQA1* | 7.11E-01 | 27% | 6% | 7.38E-08 |
| CD8^+^T cell | *TRAV10* | 6.97E-01 | 11% | 1% | 4.44E-04 |
| CD8^+^T cell | *LAIR2* | 6.51E-01 | 29% | 11% | 8.66E-05 |
| CD8^+^T cell | *CLEC2D* | 5.80E-01 | 48% | 31% | 4.66E-02 |
| CD8^+^T cell | *S100A9* | 5.49E-01 | 35% | 17% | 1.87E-02 |
| CD8^+^T cell | *SIT1* | 5.47E-01 | 25% | 9% | 3.13E-03 |
| CD8^+^T cell | *GIMAP6* | 4.81E-01 | 22% | 7% | 2.26E-03 |
| CD8^+^T cell | *MT-CO1* | 4.69E-01 | 99% | 100% | 7.72E-05 |
| CD8^+^T cell | *HLA-DQB1* | 4.61E-01 | 20% | 5% | 8.45E-04 |
| CD8^+^T cell | *CORO1A* | 4.22E-01 | 76% | 76% | 2.18E-02 |
| CD8^+^T cell | *AL157402.2* | 4.21E-01 | 14% | 3% | 4.09E-03 |
| CD8^+^T cell | *TRBV18* | 4.15E-01 | 11% | 2% | 3.65E-02 |
| CD8^+^T cell | *CISH* | 4.13E-01 | 13% | 2% | 1.62E-04 |
| CD8^+^T cell | *EIF4G2* | -2.81E-01 | 39% | 62% | 3.13E-02 |
| CD8^+^T cell | *UBC* | -2.96E-01 | 72% | 89% | 1.24E-02 |
| CD8^+^T cell | *IL2RG* | -2.98E-01 | 39% | 63% | 4.84E-02 |
| CD8^+^T cell | *SRSF5* | -3.24E-01 | 51% | 77% | 1.82E-03 |
| CD8^+^T cell | *UQCRFS1* | -3.42E-01 | 21% | 43% | 4.16E-02 |
| CD8^+^T cell | *EIF1* | -3.43E-01 | 90% | 98% | 8.82E-10 |
| CD8^+^T cell | *APMAP* | -3.56E-01 | 37% | 63% | 1.52E-02 |
| CD8^+^T cell | *ZFP36L2* | -3.60E-01 | 69% | 87% | 3.31E-02 |
| CD8^+^T cell | *TENT5C* | -3.73E-01 | 8% | 26% | 2.12E-02 |
| CD8^+^T cell | *SARAF* | -3.74E-01 | 64% | 85% | 8.72E-03 |
| CD8^+^T cell | *PPP1R15A* | -3.75E-01 | 25% | 51% | 9.24E-03 |
| CD8^+^T cell | *HLA-C* | -3.86E-01 | 88% | 97% | 2.13E-08 |
| CD8^+^T cell | *SLC44A2* | -3.86E-01 | 21% | 42% | 4.35E-02 |
| CD8^+^T cell | *CCNH* | -3.87E-01 | 17% | 38% | 1.71E-02 |
| CD8^+^T cell | *HLA-A* | -3.98E-01 | 94% | 98% | 2.01E-10 |
| CD8^+^T cell | *NFKBIZ* | -4.08E-01 | 22% | 45% | 2.64E-02 |
| CD8^+^T cell | *SRGN* | -4.13E-01 | 48% | 74% | 8.54E-04 |
| CD8^+^T cell | *RPL21* | -4.13E-01 | 65% | 77% | 6.56E-03 |
| CD8^+^T cell | *B3GNT2* | -4.13E-01 | 8% | 29% | 2.08E-03 |
| CD8^+^T cell | *RELB* | -4.21E-01 | 8% | 30% | 2.51E-03 |
| CD8^+^T cell | *CALR* | -4.30E-01 | 35% | 58% | 2.19E-02 |
| CD8^+^T cell | *PNRC1* | -4.33E-01 | 32% | 61% | 4.79E-05 |
| CD8^+^T cell | *ANKHD1* | -4.38E-01 | 12% | 34% | 2.30E-03 |
| CD8^+^T cell | *REL* | -4.43E-01 | 25% | 51% | 2.21E-03 |
| CD8^+^T cell | *DNAJC2* | -4.43E-01 | 4% | 21% | 2.11E-02 |
| CD8^+^T cell | *IFNG* | -4.45E-01 | 4% | 21% | 3.42E-02 |
| CD8^+^T cell | *PPP3CC* | -4.50E-01 | 24% | 46% | 2.38E-02 |
| CD8^+^T cell | *CSRNP1* | -4.60E-01 | 6% | 24% | 1.37E-02 |
| CD8^+^T cell | *DDOST* | -4.74E-01 | 19% | 44% | 2.08E-03 |
| CD8^+^T cell | *CD7* | -4.75E-01 | 46% | 70% | 8.10E-03 |
| CD8^+^T cell | *MAPRE2* | -4.80E-01 | 21% | 47% | 1.06E-03 |
| CD8^+^T cell | *IL2RB* | -5.01E-01 | 19% | 41% | 2.29E-02 |
| CD8^+^T cell | *EIF5A* | -5.12E-01 | 32% | 59% | 6.06E-06 |
| CD8^+^T cell | *SQSTM1* | -5.18E-01 | 35% | 58% | 8.55E-03 |
| CD8^+^T cell | *PMAIP1* | -5.43E-01 | 7% | 24% | 1.80E-02 |
| CD8^+^T cell | *BTG1* | -5.47E-01 | 69% | 93% | 7.47E-08 |
| CD8^+^T cell | *BZW1* | -5.49E-01 | 23% | 50% | 1.75E-05 |
| CD8^+^T cell | *B4GALT1* | -5.53E-01 | 15% | 38% | 2.60E-03 |
| CD8^+^T cell | *HSPA5* | -5.67E-01 | 41% | 68% | 3.93E-05 |
| CD8^+^T cell | *ATG2A* | -5.70E-01 | 5% | 30% | 6.61E-06 |
| CD8^+^T cell | *MAP3K8* | -5.74E-01 | 15% | 38% | 2.36E-03 |
| CD8^+^T cell | *TAPBP* | -5.76E-01 | 40% | 71% | 8.37E-08 |
| CD8^+^T cell | *HLA-B* | -5.77E-01 | 90% | 99% | 1.81E-21 |
| CD8^+^T cell | *NFKBIB* | -5.88E-01 | 8% | 26% | 2.45E-02 |
| CD8^+^T cell | *STK17B* | -5.89E-01 | 32% | 55% | 6.83E-03 |
| CD8^+^T cell | *HNRNPA0* | -5.91E-01 | 29% | 57% | 2.45E-06 |
| CD8^+^T cell | *MATR3.1* | -6.14E-01 | 22% | 52% | 2.78E-06 |
| CD8^+^T cell | *CLDND1* | -6.16E-01 | 12% | 31% | 2.45E-02 |
| CD8^+^T cell | *MFSD14A* | -6.24E-01 | 4% | 25% | 1.85E-04 |
| CD8^+^T cell | *RHOH* | -6.26E-01 | 22% | 48% | 4.86E-04 |
| CD8^+^T cell | *TSC22D3* | -6.41E-01 | 42% | 65% | 1.44E-03 |
| CD8^+^T cell | *NFKBIA* | -6.49E-01 | 42% | 77% | 7.99E-10 |
| CD8^+^T cell | *SBDS* | -6.67E-01 | 14% | 44% | 3.88E-07 |
| CD8^+^T cell | *JUNB* | -6.77E-01 | 57% | 83% | 6.32E-09 |
| CD8^+^T cell | *BHLHE40* | -6.98E-01 | 15% | 39% | 1.39E-04 |
| CD8^+^T cell | *PIK3R1* | -7.00E-01 | 18% | 52% | 1.05E-08 |
| CD8^+^T cell | *PCBP1* | -7.22E-01 | 34% | 64% | 1.03E-10 |
| CD8^+^T cell | *DNAJB1* | -7.36E-01 | 19% | 51% | 4.07E-09 |
| CD8^+^T cell | *ZBTB1* | -7.57E-01 | 4% | 29% | 9.80E-07 |
| CD8^+^T cell | *BTG2* | -7.79E-01 | 18% | 58% | 5.11E-12 |
| CD8^+^T cell | *DUSP2* | -7.94E-01 | 31% | 63% | 2.31E-08 |
| CD8^+^T cell | *H3F3B* | -8.03E-01 | 70% | 95% | 9.16E-25 |
| CD8^+^T cell | *CD69* | -8.54E-01 | 25% | 71% | 8.91E-17 |
| CD8^+^T cell | *NR4A2* | -9.52E-01 | 6% | 41% | 9.64E-12 |
| CD8^+^T cell | *ZFP36* | -1.01E+00 | 33% | 70% | 2.47E-14 |
| CD8^+^T cell | *DUSP1* | -1.06E+00 | 33% | 76% | 1.70E-17 |
| CD8^+^T cell | *TNFAIP3* | -1.17E+00 | 25% | 72% | 1.06E-19 |
| CD8^+^T cell | *CXCR4* | -1.19E+00 | 14% | 60% | 2.03E-18 |
| Monocyte | *HLA-DRB5* | 1.02E+00 | 69% | 41% | 1.30E-21 |
| Monocyte | *GIMAP7* | 9.31E-01 | 56% | 16% | 1.14E-35 |
| Monocyte | *RPS4Y1* | 8.88E-01 | 22% | 0% | 3.38E-21 |
| Monocyte | *HLA-DQA1* | 5.68E-01 | 60% | 30% | 2.48E-12 |
| Monocyte | *VCAN* | 5.29E-01 | 75% | 57% | 9.47E-07 |
| Monocyte | *DUSP6* | 5.11E-01 | 49% | 26% | 3.03E-09 |
| Monocyte | *MT-CO1* | 4.90E-01 | 98% | 89% | 5.59E-15 |
| Monocyte | *CYP1B1* | 4.82E-01 | 30% | 13% | 2.85E-05 |
| Monocyte | *Z93241.1* | 4.74E-01 | 50% | 28% | 1.74E-07 |
| Monocyte | *CTSD* | 4.72E-01 | 59% | 47% | 3.61E-03 |
| Monocyte | *STAB1* | 4.46E-01 | 30% | 13% | 2.68E-04 |
| Monocyte | *GIMAP4* | 4.44E-01 | 51% | 31% | 6.63E-09 |
| Monocyte | *MT-ND4* | 4.42E-01 | 97% | 90% | 5.05E-05 |
| Monocyte | *MT-CYB* | 4.20E-01 | 96% | 89% | 3.70E-09 |
| Monocyte | *NAGK* | 4.20E-01 | 59% | 44% | 1.37E-05 |
| Monocyte | *HSPA1A* | 4.15E-01 | 37% | 13% | 2.69E-11 |
| Monocyte | *ZFP36L2* | 4.13E-01 | 81% | 69% | 9.04E-06 |
| Monocyte | *IL6R* | 4.04E-01 | 43% | 26% | 2.37E-03 |
| Monocyte | *RCSD1* | 4.03E-01 | 71% | 56% | 1.10E-05 |
| Monocyte | *LYZ* | 3.87E-01 | 87% | 80% | 1.59E-05 |
| Monocyte | *CD14* | 3.82E-01 | 70% | 58% | 2.36E-02 |
| Monocyte | *TYMP* | 3.77E-01 | 88% | 77% | 8.54E-04 |
| Monocyte | *RSRP1* | 3.69E-01 | 64% | 51% | 9.70E-03 |
| Monocyte | *GIMAP1* | 3.68E-01 | 46% | 32% | 2.03E-03 |
| Monocyte | *MRPL23* | 3.60E-01 | 42% | 25% | 1.95E-04 |
| Monocyte | *TNFAIP8L2* | 3.55E-01 | 34% | 16% | 5.41E-06 |
| Monocyte | *CD1D* | 3.55E-01 | 41% | 27% | 4.51E-02 |
| Monocyte | *LMO4* | 3.54E-01 | 35% | 19% | 2.77E-03 |
| Monocyte | *OSCAR* | 3.54E-01 | 48% | 34% | 2.02E-02 |
| Monocyte | *MT-CO3* | 3.48E-01 | 98% | 92% | 5.35E-05 |
| Monocyte | *SLC16A3* | 3.36E-01 | 50% | 37% | 4.52E-03 |
| Monocyte | *GIMAP8* | 3.34E-01 | 30% | 10% | 1.36E-09 |
| Monocyte | *JAML* | 3.33E-01 | 72% | 64% | 1.42E-03 |
| Monocyte | *SLA* | 3.09E-01 | 29% | 13% | 1.05E-03 |
| Monocyte | *EVI2A* | 3.07E-01 | 33% | 20% | 4.46E-02 |
| Monocyte | *HIST1H4C* | 3.01E-01 | 46% | 32% | 4.70E-02 |
| Monocyte | *TSPO* | 3.00E-01 | 84% | 79% | 1.30E-02 |
| Monocyte | *MT-ND4L* | 2.95E-01 | 90% | 82% | 3.07E-02 |
| Monocyte | *MT-CO2* | 2.94E-01 | 99% | 94% | 2.16E-08 |
| Monocyte | *HLA-DQA2* | 2.90E-01 | 20% | 7% | 3.69E-03 |
| Monocyte | *CTSB* | 2.85E-01 | 74% | 67% | 2.86E-02 |
| Monocyte | *CCR2* | 2.81E-01 | 19% | 5% | 1.05E-06 |
| Monocyte | *CCDC88A* | 2.80E-01 | 53% | 36% | 8.22E-03 |
| Monocyte | *MT-ND5* | 2.77E-01 | 93% | 80% | 5.59E-03 |
| Monocyte | *SLC35E2B* | 2.70E-01 | 21% | 8% | 1.84E-03 |
| Monocyte | *RPL6* | 2.68E-01 | 90% | 82% | 2.20E-06 |
| Monocyte | *ATP6V1B2* | 2.61E-01 | 48% | 32% | 5.32E-03 |
| Monocyte | *RPS27* | -2.51E-01 | 89% | 88% | 6.42E-03 |
| Monocyte | *ATF3* | -2.63E-01 | 24% | 44% | 1.21E-03 |
| Monocyte | *BTG2* | -2.65E-01 | 55% | 71% | 2.77E-02 |
| Monocyte | *TNF* | -2.65E-01 | 14% | 32% | 1.25E-03 |
| Monocyte | *C15orf48* | -2.77E-01 | 0% | 11% | 1.89E-04 |
| Monocyte | *NXT1* | -2.83E-01 | 23% | 39% | 3.32E-02 |
| Monocyte | *ARL4A* | -2.87E-01 | 11% | 32% | 5.45E-06 |
| Monocyte | *YBX1* | -2.98E-01 | 70% | 77% | 1.09E-02 |
| Monocyte | *MAPK1IP1L* | -3.02E-01 | 26% | 45% | 9.12E-03 |
| Monocyte | *BBC3* | -3.06E-01 | 7% | 25% | 7.25E-06 |
| Monocyte | *CLEC2B* | -3.16E-01 | 33% | 48% | 3.45E-02 |
| Monocyte | *HLA-A* | -3.17E-01 | 94% | 95% | 8.60E-08 |
| Monocyte | *ZC3H12A* | -3.24E-01 | 6% | 24% | 5.44E-06 |
| Monocyte | *PFKFB3* | -3.32E-01 | 7% | 26% | 2.52E-06 |
| Monocyte | *MAP1LC3B* | -3.33E-01 | 58% | 73% | 2.60E-03 |
| Monocyte | *PCIF1* | -3.37E-01 | 21% | 36% | 3.20E-02 |
| Monocyte | *HBEGF* | -3.40E-01 | 3% | 19% | 2.81E-06 |
| Monocyte | *OSER1* | -3.49E-01 | 21% | 36% | 3.04E-02 |
| Monocyte | *MAP3K8* | -3.51E-01 | 40% | 61% | 1.57E-04 |
| Monocyte | *CDKN1A* | -3.53E-01 | 37% | 62% | 5.80E-07 |
| Monocyte | *B3GNT2* | -3.55E-01 | 12% | 26% | 3.80E-02 |
| Monocyte | *B2M* | -3.58E-01 | 98% | 100% | 1.92E-09 |
| Monocyte | *OSM* | -3.73E-01 | 5% | 18% | 1.52E-03 |
| Monocyte | *DDIT4* | -3.94E-01 | 19% | 39% | 3.10E-04 |
| Monocyte | *GABARAPL1* | -3.96E-01 | 18% | 36% | 1.57E-03 |
| Monocyte | *MIR22HG* | -4.00E-01 | 17% | 33% | 1.97E-02 |
| Monocyte | *RBKS* | -4.05E-01 | 14% | 31% | 2.49E-03 |
| Monocyte | *HLA-B* | -4.09E-01 | 98% | 99% | 2.33E-21 |
| Monocyte | *MCL1* | -4.15E-01 | 64% | 78% | 2.36E-04 |
| Monocyte | *FOS* | -4.15E-01 | 87% | 90% | 5.11E-08 |
| Monocyte | *PPP1R15A* | -4.22E-01 | 70% | 82% | 7.54E-11 |
| Monocyte | *STX11* | -4.22E-01 | 38% | 55% | 1.82E-03 |
| Monocyte | *AP001189.1* | -4.30E-01 | 0% | 8% | 2.59E-02 |
| Monocyte | *ID2* | -4.30E-01 | 36% | 54% | 5.21E-04 |
| Monocyte | *H3F3B* | -4.32E-01 | 94% | 95% | 4.92E-08 |
| Monocyte | *KLF6* | -4.35E-01 | 74% | 83% | 3.06E-05 |
| Monocyte | *JUNB* | -4.56E-01 | 90% | 90% | 1.08E-12 |
| Monocyte | *YPEL5* | -4.59E-01 | 40% | 57% | 3.41E-03 |
| Monocyte | *RASSF5* | -4.60E-01 | 26% | 45% | 4.27E-03 |
| Monocyte | *NINJ1* | -4.63E-01 | 53% | 65% | 8.24E-03 |
| Monocyte | *PER1* | -4.66E-01 | 11% | 38% | 1.74E-11 |
| Monocyte | *DDIT3* | -4.73E-01 | 13% | 41% | 4.57E-11 |
| Monocyte | *SQSTM1* | -4.77E-01 | 63% | 76% | 1.75E-04 |
| Monocyte | *CSRNP1* | -4.82E-01 | 27% | 53% | 4.09E-09 |
| Monocyte | *STK17B* | -4.88E-01 | 49% | 69% | 9.58E-08 |
| Monocyte | *RELB* | -4.92E-01 | 31% | 57% | 9.90E-07 |
| Monocyte | *LYN* | -4.95E-01 | 57% | 72% | 8.07E-06 |
| Monocyte | *PNRC1* | -5.09E-01 | 55% | 70% | 3.59E-06 |
| Monocyte | *DUSP1* | -5.11E-01 | 89% | 89% | 6.29E-16 |
| Monocyte | *IER3* | -5.24E-01 | 24% | 44% | 2.18E-06 |
| Monocyte | *GLUL* | -5.61E-01 | 40% | 59% | 1.12E-02 |
| Monocyte | *SAT1* | -5.63E-01 | 92% | 96% | 5.46E-15 |
| Monocyte | *SRGN* | -5.79E-01 | 89% | 95% | 5.26E-11 |
| Monocyte | *PHACTR1* | -5.80E-01 | 27% | 45% | 1.92E-04 |
| Monocyte | *PDE4B* | -5.83E-01 | 13% | 38% | 1.15E-09 |
| Monocyte | *THBS1* | -5.86E-01 | 5% | 17% | 5.58E-03 |
| Monocyte | *NR4A1* | -5.94E-01 | 24% | 54% | 2.50E-13 |
| Monocyte | *NFKBIZ* | -5.96E-01 | 62% | 80% | 7.82E-10 |
| Monocyte | *SAMSN1* | -6.00E-01 | 16% | 34% | 1.68E-03 |
| Monocyte | *RGS2* | -6.02E-01 | 49% | 71% | 3.06E-10 |
| Monocyte | *KLF10* | -6.11E-01 | 47% | 67% | 6.65E-11 |
| Monocyte | *PLEK* | -6.19E-01 | 62% | 81% | 8.60E-16 |
| Monocyte | *PLAUR* | -6.21E-01 | 64% | 78% | 2.76E-09 |
| Monocyte | *PIM3* | -6.29E-01 | 17% | 51% | 5.27E-19 |
| Monocyte | *ATP2B1-AS1* | -6.39E-01 | 55% | 74% | 1.15E-08 |
| Monocyte | *NAMPT* | -6.45E-01 | 50% | 75% | 5.09E-10 |
| Monocyte | *ICAM1* | -6.54E-01 | 24% | 50% | 7.48E-10 |
| Monocyte | *IFITM3* | -6.59E-01 | 68% | 82% | 2.42E-05 |
| Monocyte | *PCBP1* | -6.67E-01 | 61% | 77% | 4.73E-17 |
| Monocyte | *BHLHE40* | -6.77E-01 | 18% | 46% | 3.70E-12 |
| Monocyte | *TMEM176B* | -6.88E-01 | 32% | 64% | 1.52E-15 |
| Monocyte | *TNFAIP3* | -7.04E-01 | 34% | 60% | 2.86E-12 |
| Monocyte | *SGK1* | -7.20E-01 | 20% | 51% | 1.36E-14 |
| Monocyte | *TMEM176A* | -7.30E-01 | 22% | 56% | 5.74E-17 |
| Monocyte | *ABCA1* | -7.41E-01 | 22% | 49% | 2.37E-10 |
| Monocyte | *FTH1* | -7.53E-01 | 97% | 100% | 5.78E-37 |
| Monocyte | *CD83* | -7.70E-01 | 36% | 69% | 2.51E-20 |
| Monocyte | *CCL4L2* | -7.79E-01 | 0% | 13% | 4.57E-06 |
| Monocyte | *HIF1A* | -8.03E-01 | 30% | 67% | 6.24E-23 |
| Monocyte | *SOD2* | -8.08E-01 | 56% | 78% | 6.80E-15 |
| Monocyte | *CCL5* | -8.21E-01 | 6% | 23% | 9.44E-06 |
| Monocyte | *DUSP2* | -8.51E-01 | 26% | 55% | 6.48E-14 |
| Monocyte | *IL1B* | -8.90E-01 | 12% | 38% | 1.14E-11 |
| Monocyte | *CXCR4* | -8.91E-01 | 15% | 43% | 3.94E-13 |
| Monocyte | *BCL2A1* | -9.71E-01 | 37% | 62% | 5.05E-12 |
| Monocyte | *TUBB1* | -9.74E-01 | 2% | 13% | 4.93E-04 |
| Monocyte | *MYL9* | -1.00E+00 | 3% | 15% | 3.17E-03 |
| Monocyte | *CDKN2D* | -1.05E+00 | 21% | 58% | 5.16E-23 |
| Monocyte | *NFKBIA* | -1.06E+00 | 81% | 89% | 2.10E-31 |
| Monocyte | *IFITM2* | -1.06E+00 | 84% | 90% | 4.12E-05 |
| Monocyte | *CCL3* | -1.08E+00 | 17% | 44% | 5.38E-12 |
| Monocyte | *ZFP36* | -1.13E+00 | 73% | 90% | 9.32E-50 |
| Monocyte | *CAVIN2* | -1.14E+00 | 4% | 17% | 7.54E-04 |
| Monocyte | *CXCL8* | -1.18E+00 | 21% | 49% | 1.56E-13 |
| Monocyte | *IFITM1* | -1.23E+00 | 18% | 38% | 3.90E-06 |
| Monocyte | *CCL3L1* | -1.29E+00 | 9% | 47% | 1.28E-24 |
| Monocyte | *PPBP* | -1.83E+00 | 7% | 30% | 1.36E-10 |
| Monocyte | *G0S2* | -2.01E+00 | 13% | 66% | 2.14E-46 |
| NK cell | *MYOM2* | 1.21E+00 | 79% | 29% | 9.08E-05 |
| NK cell | *GIMAP7* | 9.16E-01 | 86% | 58% | 3.47E-02 |
| NK cell | *RPL21* | -5.92E-01 | 93% | 98% | 1.41E-02 |

**Table S4. Cell-cell interactions in female AS.**

| Computed significant cell-cell interaction (subset) | | | |
| --- | --- | --- | --- |
|  | Secreted Signaling (SS) | ECM-Receptor (EMCr) | Cell-Cell Contact (CCC) |
| Up-regulated in AS | VEGI | VTN | CD46, NEGR |
| Down-regulated in AS | *CALCR, CSF3, FGF, HGF, IGF, IL4, LIFR, NPR2, SEMA3, WNT* | *COLLAGEN, VWF* | *ESAM, JAM, NECTIN, SELL, VISTA* |
| Overlapped in HC and AS | *ACTIVIN, ANNEXIN, APRIL, BAFF, BAG, BMP, BTLA, CCL, CD30, CD40, COMPLEMENT, CSF, CXCL, EGF, FASLG, FLT3, GALECTIN, GAS, GH, GRN, IFN-II, IL1, IL10, IL16, IL2, IL6, LIGHT, LT, MIF, NRG, OSM, PARs, PDGF, RESISTIN, TGFb, TNF, TRAIL, VEGF* | *FN1, THBS* | *ADGRE5, ALCAM, APP, CD22, CD226, CD23, CD40, CD45, CD48, CD6, CD80, CD86, CD96, CD99, CDH, CDH1, CLEC, EPHA, EPHB, GP1BA, ICAM, ITGB2, L1CAM, LCK, MHC-I, MHC-II, MPZ, NCAM, NOTCH, OCLN, PECAM1, PVR, SELPLG, SEMA4, SEMA7, TIGIT* |

**Figure S1. Multimodal single-cell analyses workflow.**


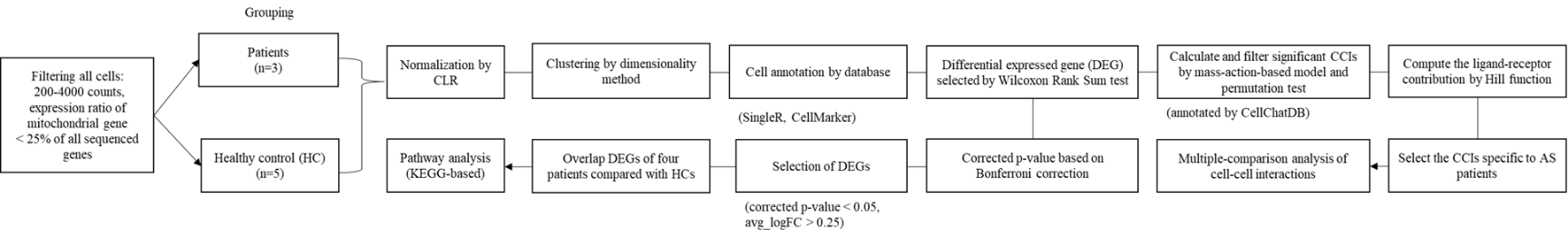


**Figure S1.** Multimodal single-cell analyses including single-cell-level unbiased transcriptome, surface protein expression, pseudotemporal trajectory analysis, cell-cell interaction analysis, and T-cell receptor repertoire were shown in this analysis workflow.

**Figure S2. Expression of *NFKBIA* in the T-cell subpopulations from male AS patients.**

**
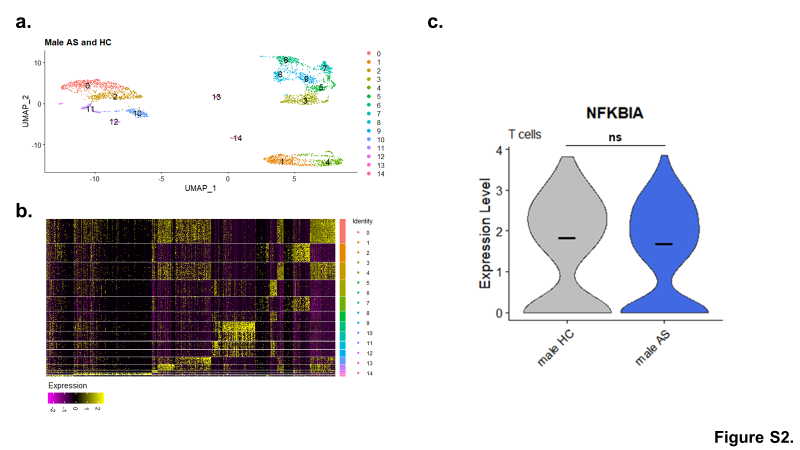
**

**Figure S2. Expression of *NFKBIA* in the T-cell subpopulations from male AS patients.**

(a) Cell sub-populations in the PBMCs from male AS patients and gender-matched healthy controls were visualized with uniform manifold approximation and projection (UMAP). (b) Heatmap of specific gene expression profile of each cell population in the PBMCs from male AS patients and gender-matched healthy controls. (c) Comparing cell-type transcriptional expression in the T-cell populations (including T_1 and T_2) between male AS patients (n=4) group and the gender-matched healthy controls (n=5) group.

**Figure S3. High-throughput pairing of entire T-cell receptor alpha and beta sequences from female-AS-specific T cells through single-cell TCR-sequencing.**

**
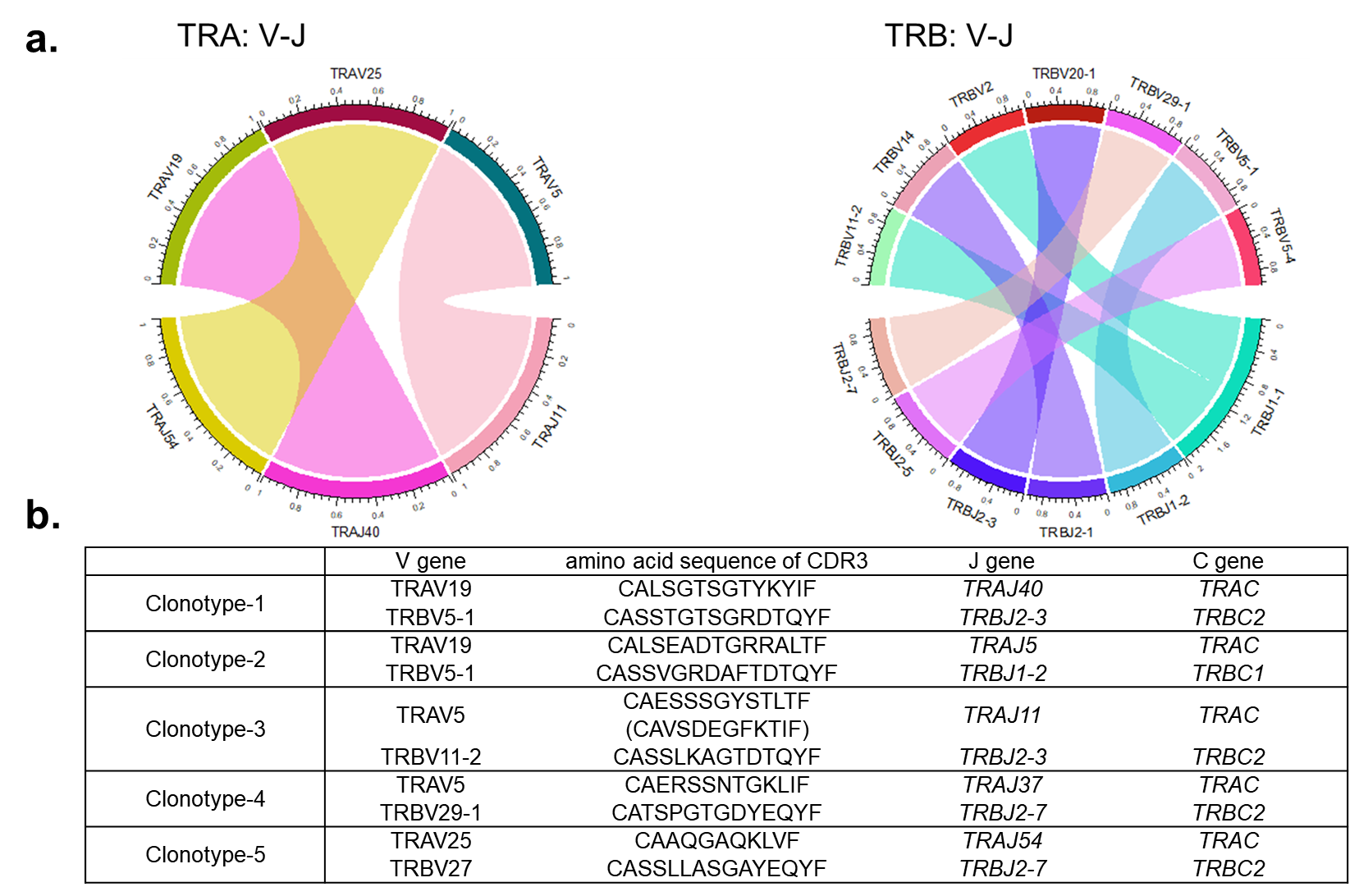
**

**Figure S3. High-throughput pairing of entire T-cell receptor alpha and beta sequences from female-AS-specific T cells through single-cell TCR-sequencing.**

The T-cell receptors (TCRs) of the female-AS-specific T cells of the female AS patients were identified. (a) V-J recombination of alpha-chain and beta-chain of the paired TCRs of the female-AS-specific T cells from the female AS patients. Three types of V-J recombination were found in the alpha-chain: TRAV19-TRAJ40, TRAV25-TRAJ54, and TRAV5-TRAJ11. Seven types of V-J recombinations were found in the beta-chain: TRBV11-2-TRBJ1-1, TRBV14-TRBJ2-1, TRBV2-TRBJ1-1, TRBV20-1-TRBJ2-3, TRBV29-1-TRBJ2-7, TRBV5-1-TRBJ1-2, and TRBV5-4-TRBJ2-5. (b) V-J-C recombination and CDR3 amino acid sequences of the paired TCRs of the female-AS-specific T cells of the female AS patients.

**Figure S4. Identifying female-AS-specific cell-cell communications.**

**
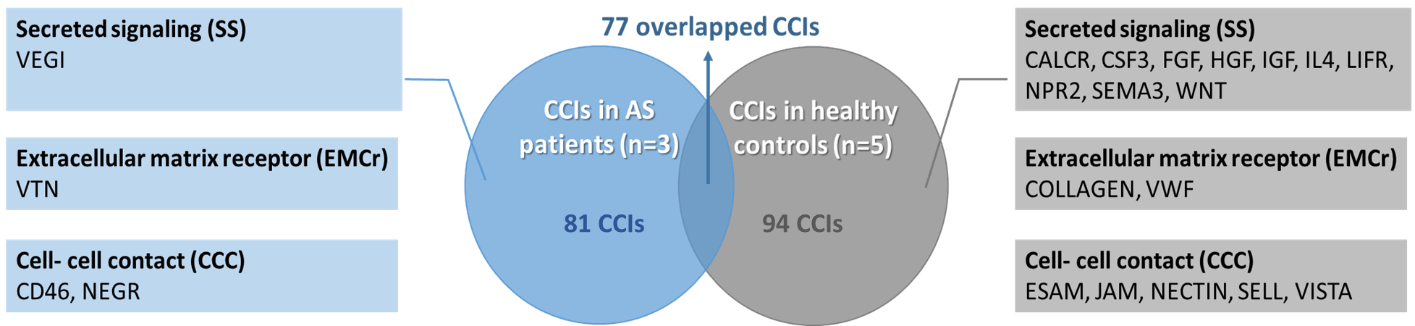
**

**Figure S4. Identifying female-AS-specific cell-cell communications.** By computing the significant cell-cell interactions (CCIs) in the PBMCs, four CCIs were identified in female AS patients, VEGI was found in the secreted signaling pathway (SS), VTN in the extracellular receptor (EMCr), and CD46 and NEGR in cell-cell contacts (CCC).

**Figure S5. Transcriptomic profiling of GIMAP7^+^NFKBIA^-^ T cells in female AS.**


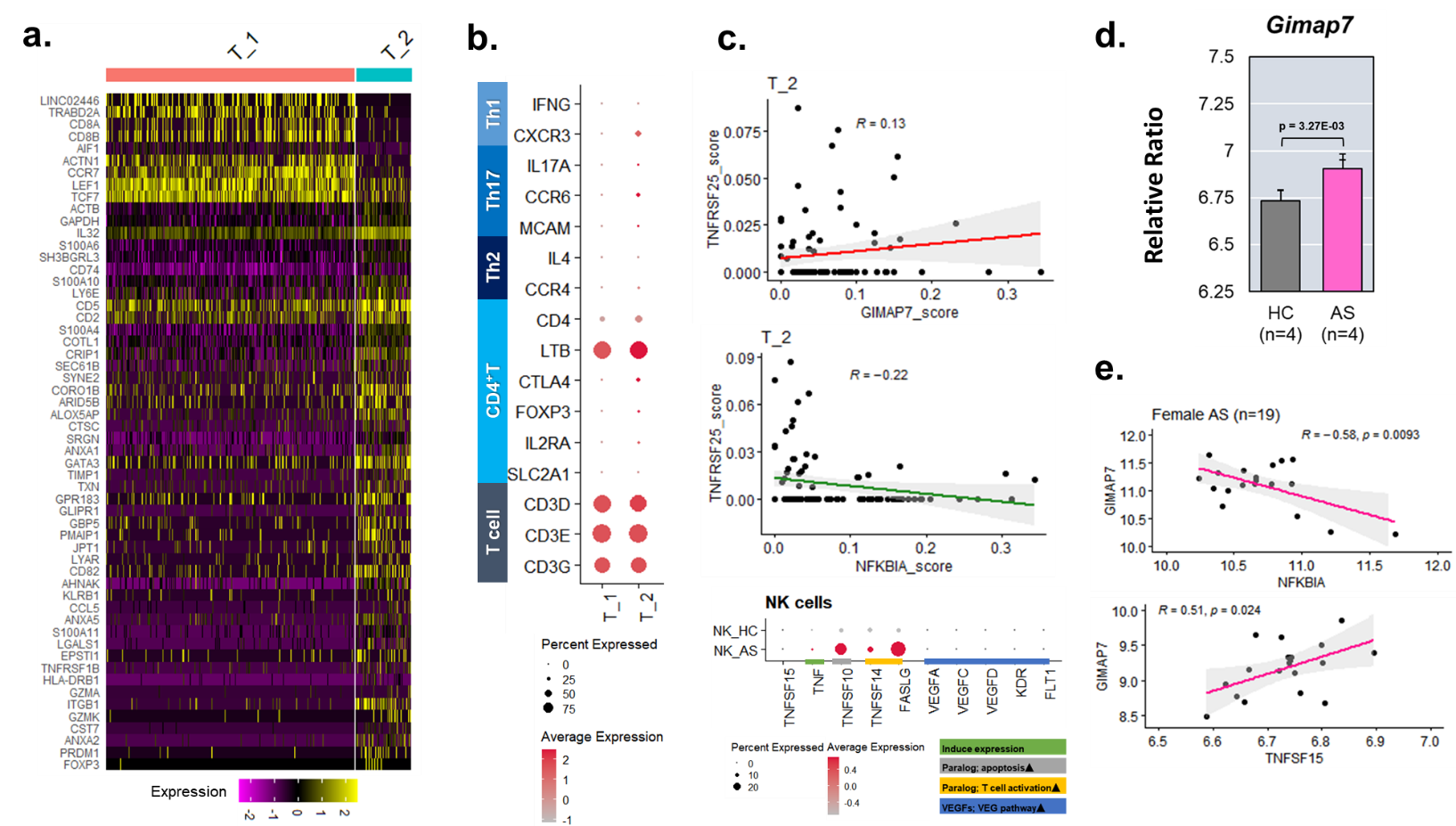


**Figure S5. Transcriptomic profiling of GIMAP7^+^NFKBIA^-^ T cells in female AS.**

(a) Heatmap of the marker genes corresponding to each cluster are identified, which demonstrated that there was distinct gene expression profiling between cells of T_1 and T_2. (b) Dot plot shows the expression profiles of marker genes of Th1, Th17, Th2, and CD4^+^ T cell in T_1 and T_2 cells. (c) To determine the correlation between NK cells and T cells in female AS, co-expression analysis of the female-AS-specific T-cell genes (including *GIMAP7* and *NFKBIA*) and VEGI genes (*TNFRSF25*, receptor of VEGI) was performed. To identify the contribution of inhibiting vascular endothelial growth (VEG) of the NK cells from female patients with AS, the expression profiles of genes involving VEG were compared with those of healthy controls. The genes involve inhibiting VEG, *TNFSF15* (also known as VEGI), *TNF* (inducer of VEGI), *TNFSF10* (paralog of VEGI, promoting apoptosis), *TNFSF14*, and *FASLG* (paralog of VEGI, promoting T cell activation) were up-regulated in NK cells of patients. The genes involve promoting VEG, *VEGFA*, *VEGFC*, *VEGFD*, *KDR*, and *FLT1* showed no difference between the patients and healthy controls. (d) To validate female-AS-specific T cell characteristics, public datasets from the SpA mouse model were analyzed. Compared with the control mouse model, spines from AS mouse model (PGISp mice) also had higher expression level of *Gimap7*. (e) To validate female-AS-specific T cell characteristics, public datasets from human female patients were analyzed. Compared to the female healthy controls, both significant negative expression correlation of *GIMAP7* and *NFKBIA*, and the positive expression correlation of *GIMAP7* and *TNFSF15* were only found in the female patients.

**Supplementary Methods**

**Ethical statement**

This investigation (CE19024B) was approved by the Institutional Review Board and the Ethics Committee of the Taichung Veterans General Hospital, Taichung, Taiwan. Written informed consent was obtained from the subjects or their family members in accordance with the institutional requirements and Declaration of Helsinki principles.

**Patient**

The diagnosis of AS conformed to European League Against Rheumatism (EULAR) criteria^1^. Three average 34-year-old female AS patients with average patient global score of 2.3 and average physician global score of 2.3 was assessed. The average C-reactive protein (CRP, mg/dL) of the patients was 0.6; whereas, the average erythrocyte sedimentation rate before and after treatment (ESR, mm/h) was 14.7. Four average 25-year-old male AS patients with the average patient and physician global score of 3.5 were evaluated. The patients' average C-reactive protein (CRP, mg/dL) was 1.991; whereas the average erythrocyte sedimentation rate before and after treatment (ESR, mm/h) was 20 (**Table S1**). Peripheral blood mononuclear cell samples (PBMCs) of female and male AS patients and healthy donors were isolated using SepMate™.

**Single-cell RNA-seq for PBMCs obtained from the AS patients**

Samples of peripheral blood mononuclear cells (PBMCs) were isolated using SepMate™. The barcoded antibodies of TotalSeq™-C0251, TotalSeq™-C0252, TotalSeq™-C0253, TotalSeq™-C0254, and TotalSeq™-C0255 were used to label each sample for sample multiplexing.

These antibodies recognized human CD298 and β2 microglobulin, and labelled cells. Antibody-labelled sample were loaded into a 10x Genomics microfluidics chip and encapsulated with barcoded hashtag-oligos (HTO)-containing gel beads using the 10x Genomics chromium controller^2^. The captured mRNAs were barcoded, converted to barcoded cDNA, and pooled into scRNA-seq libraries for Illumina sequencing.

**scTCR-seq analysis of V(D)J genes and sequences of CDR3 region**

TCR sequences of all T cells from each patient and healthy donor were collected using the Chromium Single-Cell V(D)J Enrichment Kit (10x Genomics) and the manufacturer’s instructions. The CDR3 region was identified using the cellranger vdj pipeline (v3.1.0). The output matrix of scTCR-seq contained clonotype, frequency, CDR3 variable region sequence, and cell barcode information. The amino acid sequences and V(D)J composition of each TCR of the three female AS patients were assembled by integrating the scTCR-seq matrix with cell barcodes. The visualizations of alpha- and beta-train of TCRs were accomplished via chordDiagram function of the R package circlize.

**Surface protein gene expression quantification via CITE-seq**

For quantifying surface protein gene expression of each cell, the samples were proceeded with CITE-seq (Cellular Indexing of Transcriptomes and Epitopes by Sequencing). A pool of TotalSeq antibodies was added at 1 μg each TotalSeq-C0007 anti-human CD274 (B7-H1, PD-L1) antibody, TotalSeq-C0053 anti-human CD11c antibody, TotalSeq™-C0063 anti-human CD45RA antibody, TotalSeq-C0072 anti-human CD4 antibody, TotalSeq-C0080 anti-human CD8a antibody, TotalSeq-C0083 anti-human CD16 antibody, TotalSeq-C0084 anti-human CD56 (NCAM) recombinant, TotalSeq-C0085 anti-human CD25 antibody, TotalSeq™-C0087 anti-human CD45RO antibody, TotalSeq-C0088 anti-human CD279 (PD-1) antibody, TotalSeq-C0159 anti-human HLA-DR antibody, TotalSeq-C0180 anti-human CD24 antibody and TotalSeq-C0390 anti-human CD127 (IL-7Rα) antibody, and thereafter, the cells were incubated for 30 min at 4 °C, followed by washing thrice in staining buffer. Libraries were constructed according to the manufacturer’s protocol, with the following modifications according to the CITE-Seq protocol (<https://cite-seq.com/protocol>).

**scRNA-seq data analysis**

The raw reads were mapped to the human genome reference GRCh38, demultiplexed and barcoded by cellranger count commend of Cell Ranger Software Suite (Version 3.1.0) (<https://support.10xgenomics.com>). In the end, a gene-barcode matrix contained strand-specific alignment to exonic sequence, gene identifiers, cell-identifying barcodes and unique molecular identifier (UMI). The gene-barcode matrix enclosed gene counts from each aggregated sample, and was demultiplexed by CITE-Seq-Count 1.4.3 (<https://github.com/Hoohm/CITE-seq-Count>)^2^. In order to visualize individual samples from different batches, the generated gene matrix of Cell Ranger and CITE-Seq-Count were proceeded via Seurat v3 (<https://satijalab.org/>)^2, 3^. Thereafter, the criteria for quality control was applied to each cell: the normal distribution of feature gene count ranged from 200 to 4000, the expression percentage of mitochondrial genes was less than 25%, and the doublet and negative cells were removed by hashtag information. Following PBMC datasets integration (8 samples from three female patients and five gender-matched healthy donors; 9 samples from four male patients and five gender-matched healthy donors), a total of 2,354 female cohort cells (824 cells for the three female-AS patients; 1,530 cells for the gender-matched healthy controls) and 3,302 cells of the male cohort (2,029 cells for the three male AS patients; 1,273 cells for the gender-matched healthy controls) were left for analysis. The filtered PBMC datasets were integrated with IntegrateData function based on the anchors between these datasets via calculation of function FindIntegrationAnchors for batch effect correction. In addition, PBMC datasets of female and male subjects were integrated respectively. The data was normalized using log-normalization and clustered via dimensionality-reduction method and the FindClusters function (resolution parameter=0.5) in the R package Seurat v3. Clusters were annotated by cell-classifier SingleR^4^ and validated using the human database of CellMarker (<http://biocc.hrbmu.edu.cn/CellMarker/>) and SignacX^5^. For example, in female-AS patients, there were fewer CD8^+^ T cells [CD8^+^T_1 cells:17.8% (HC) 🡪 10.3% (fAS)], fewer NK cells [NK_1 cells: 12.4% (HC) 🡪 3.4% (fAS)] and more CD4^+^ T cells [T_2 cells: 3.4% (HC) 🡪 4.6% (fAS)]. The study analysis generated 15 cell clusters, categorized into the following six major cell types according to cell-type markers: monocytes (*LYZ*), CD4^+^ T cells (*CD3D*, *CD3E*, *CD4*), CD8^+^ T cells (*CD3D*, *CD3E*, *CD8*), B cells (*CD79A*), and NK cells (*KLRF1*) (**Figure** **1d**). To determine the differences between T_1 and T_2, we performed heatmap analysis and found distinct gene expression pattern between T_1and T_2. For identifying female-AS-cell-type-specific features, we planned a multiple filtering strategy: for each cell type, we collected the significant DEGs (p<0.05) between each female AS patient and healthy donors and selected the overlapped DEGs. To examine the disease-associated expression features, differential expression analysis was performed to determine the significance-filtered differentially expressed genes (DEGs) of each cell population in PBMCs from the AS patients and the gender-matched healthy controls. The *GIMAP7*^+^*NFKBIA*^-^ T cells were CD4 dominant T-cell populations. Specifically, there were 22% CD4 memory T cells, 49% CD4 naïve T cells, and <1% Treg cells in the *GIMAP7*^+^*NFKBIA*^-^ T cells. Gene Ontology Biological Process (GOBP) analysis^6^ from DEGs was performed using g:Profiler (<http://biit.cs.ut.ee/gprofiler/gost>) and ShinyGO (<http://bioinformatics.sdstate.edu/go/>), which also performs functional enrichment analysis known as over-representation analysis (ORA) or gene set enrichment analysis.

**Pseudotemperal ordering of cells analysis**

In order to infer the pseudotemperal trajectory of cells, identifying the genes that significantly dominated the cell differentiation, data re-ordering and re-dimensionality-reduction were proceed via Monocle v2^7^. After normalization and dispersion-estimation, the potential ordering genes were identified and filtered out by excluding the low-quality cells (e.g. empty droplets, doublets) and low-expressed genes (< 10% cells expressed). The filtered data was divided into two groups: patients (n=3) and healthy controls (n=5). And differential expression analysis selected the significant ordering genes (q-value < 0.01). After extracting the expression profile of the significant ordering genes, the cell trajectory state was determined via dimensionality-reduction based on discriminative dimensionality reduction with trees (DDRTree) and re-ordering data by encoding where each cell maps to the trajectory. Eventually, the visualization of pseudotemperal cell trajectory highlighted the direction and states of cell differentiation.

**Cell-cell communication analysis**

To identify the communications between cells, we extracted expression profiles of the overexpressed genes of ligands and receptors of the signaling pathways via CellChat^8^. The expression profiles of overexpressed cell-cell communications were calculated interaction probability, projected onto protein-protein interaction networks. The significant communications were filtered out by excluding the interactions that only presented in few certain cell groups (<10 cells), inferred interaction pathway probability, and aggregated into cell-cell interaction networks by counting the number of link and/or summarizing the communication probability. In the end, the visualization of cell-cell communications demonstrated the involved cells, roles of each cell group and ligand-receptor contribution of certain interaction. Among the four CCIs identified in patients, the one with the highest T cell participation compared to other cell populations and transmitted by T_1 and/or T_2 cells was selected. Among the four female-AS-specific CCIs, only the secreted signaling communication of vascular endothelial growth inhibitor (VEGI) was found to be transmitted by T_2 cells and considered a potential signaling channel of the female-AS-specific T cells.

**Validation analysis on the genomic targets of female-AS based on the public transcriptomic datasets**

The public microarray datasets of female proteoglycan-induced spondylitis (PGISp) mouse spines were deposited in the NCBI GEO database (GSE41039) by K. R. Haynes, et al.^9^ Microarray datasets were assigned to wild-type mice groups (n=4) and PGISp mice (n=4), and the gene expression profile was visualized using the NCBI website tool GEO2R. The public microarray datasets of female patients and healthy controls were deposited in the NCBI GEO database (GSE73754) by E. Gracey et al.^10^ The datasets were assigned to healthy controls (n=10) and HLA-B27-positive patients (n=19), acquired the expression profiles of genes of interest via GEO2R, and visualized correlation of gene expression via ggscatter function from R-package ggpubr.

**Supplementary References**

1. Braun J*, et al.* 2010 update of the ASAS/EULAR recommendations for the management of ankylosing spondylitis. *Annals of the Rheumatic Diseases* **70**, 896 (2011).

2. Stoeckius M*, et al.* Cell Hashing with barcoded antibodies enables multiplexing and doublet detection for single cell genomics. *Genome Biology* **19**, 224 (2018).

3. Butler A, Hoffman P, Smibert P, Papalexi E, Satija R. Integrating single-cell transcriptomic data across different conditions, technologies, and species. *Nature Biotechnology* **36**, 411-420 (2018).

4. Aran D*, et al.* Reference-based analysis of lung single-cell sequencing reveals a transitional profibrotic macrophage. *Nature Immunology* **20**, 163-172 (2019).

5. Chamberlain M, Hanamsagar R, Nestle FO, de Rinaldis E, Savova V. Cell type classification and discovery across diseases, technologies and tissues reveals conserved gene signatures and enables standardized single-cell readouts. *bioRxiv*, 2021.2002.2001.429207 (2021).

6. Consortium GO. The gene ontology resource: 20 years and still GOing strong. *Nucleic acids research* **47**, D330-D338 (2019).

7. Qiu X, Hill A, Packer J, Lin D, Ma Y-A, Trapnell C. Single-cell mRNA quantification and differential analysis with Census. *Nature Methods* **14**, 309-315 (2017).

8. Braun J, Sieper J. Ankylosing spondylitis. *The Lancet* **369**, 1379-1390 (2007).

9. Haynes KR*, et al.* Excessive bone formation in a mouse model of ankylosing spondylitis is associated with decreases in Wnt pathway inhibitors. *Arthritis Research & Therapy* **14**, R253 (2012).

10. Gracey E*, et al.* Sexual Dimorphism in the Th17 Signature of Ankylosing Spondylitis. *Arthritis & Rheumatology* **68**, 679-689 (2016).
